# Emotional Labor, Gendered Care, and Educational Leadership Educators During the COVID-19 Pandemic

**DOI:** 10.3390/bs16030324

**Published:** 2026-02-26

**Authors:** Jill Channing, Georgina E. Wilson

**Affiliations:** 1 Department of Educational Leadership & Policy, East Tennessee State University, Johnson City, TN 37614, USA; 2 Department of Counseling, Educational Leadership, and Higher Education, Central Michigan University, Mount Pleasant, MI 48859, USA; wilso2ga@cmich.edu

**Keywords:** emotional labor, care ethics, educational leadership, faculty well-being, gendered labor, COVID-19, higher education

## Abstract

The COVID-19 pandemic intensified faculty emotional labor as instructors were expected to sustain learning while responding to students’ grief, isolation, and uncertainty. Educational leadership educators occupy a distinctive role as mentors and models for current and aspiring PK–12 and higher education leaders. Using a secondary phenomenological analysis, we reanalyzed de-identified Zoom interview transcripts (2022) from nine U.S. educational leadership educators (seven women; four educators of color) originally collected to examine caring pedagogies. Guided by Hochschild’s emotional labor theory and feminist care ethics, with particular attention to Tronto’s political theory of care, we conducted a theoretically informed thematic analysis focused on caring expectations, role boundaries, and well-being. Findings highlight five interrelated themes: serving as an “anchor” during crisis; blurred instructional–counseling roles and invisible care work; gendered and racialized expectations of availability; competing care obligations across work and home; and boundary-setting as resistance and sustainability. Participants described deep relational commitments to students alongside exhaustion, role strain, and frustration with institutional cultures that assumed limitless capacity to care without reciprocal support. Emotional labor in leadership education should be recognized as central leadership work, and sustainable cultures of care require systemic policies that redistribute and resource care labor.

## 1. Introduction

### 1.1. Introduction

The COVID-19 pandemic disrupted higher education on a global scale, reshaping where and how teaching occurred and intensifying concerns about stress, burnout, and mental health among faculty and students. Studies across disciplines have documented sharp increases in workload, emotional exhaustion, and uncertainty as faculty redesigned courses, adapted to remote modalities, and responded to students’ personal crises (Constantin et al., 2024; Heath & Holyoke, 2023; Newcomb, 2021; Pereira, 2021). In graduate and professional programs, these pressures were compounded by the dual roles of many students as practicing professionals navigating crisis conditions in their own workplaces (Curran et al., 2023; Matheson et al., 2019). While this scholarship has illuminated the pressures of pandemic-era teaching, less attention has been paid to the emotional labor embedded in faculty roles, particularly the invisible work of soothing anxiety, absorbing frustration, and providing individualized care to students in distress. Hochschild’s (1983/2012) theorization of emotional labor as the management of one’s own feelings and the orchestration of others’ emotions under organizational “feeling rules” has been widely applied in health and care professions (Curran et al., 2023; Lim et al., 2018; Mariyanti et al., 2025; Pan et al., 2019; Topuz et al., 2024). Yet similar processes in higher education—often enacted in emails, office hours, feedback conversations, and crisis response—remain under-recognized despite their importance for student engagement and persistence (Constantin et al., 2024; Newcomb, 2021).

Educational leadership educators occupy a particularly intricate position within this landscape. They prepare current and aspiring principals, superintendents, and higher education leaders to navigate complexity, respond ethically to crises, and cultivate supportive organizational cultures (Azzahra & Rahmawati, 2024; Smit, 2018). Their courses frequently foreground ethics, social justice, relational leadership, and care, positioning them as both scholars of leadership and models of leadership practice. During the COVID-19 pandemic, these educators were not only redesigning their own courses; they were also helping their students—many of whom were PK–12 and higher education leaders—to guide schools and colleges through unprecedented disruption (Dopelt et al., 2021; Mafratoğlu et al., 2025). As a result, educational leadership educators stood at the nexus of multiple layers of crisis: their own institutions, their students’ institutions, and their students’ personal and family lives.

In earlier work, we analyzed this same qualitative dataset to understand how educational leadership educators cultivated cultures of care in their classrooms during the pandemic, focusing on caring spaces, relationships, rigor and flexibility, trauma-informed pedagogy, and wellness practices (Channing & Wilson, 2023). That analysis highlighted the ways educators integrated ethics of care into curriculum and pedagogy but treated care primarily as a positive, productive practice—a resource for student learning and moral development (Channing & Wilson, 2023). The present article extends this work by posing a different question:

How did educational leadership educators experience and negotiate the emotional labor involved in providing care to students during the COVID-19 pandemic?

Rather than centering the benefits of care for students, we foreground the costs and consequences of caring for educators themselves. We draw on Hochschild’s (1983/2012) theory of emotional labor, feminist analyses of gendered and racialized academic labor (N. N. Johnson, 2021; Pereira, 2021; Scolnic, 2024), and Tronto’s (1993, 2013) political theory of care to understand how pandemic conditions intensified existing patterns of invisible, often feminized and racialized, care work in higher education. Phenomenological and relational approaches to leadership inquiry (Gilstrap, 2007; Guerra, 2014; Smit, 2018) further guide our attention to lived experience, narrative, and relational dynamics among educational leadership educators.

Using a secondary phenomenological analysis of interviews with nine educational leadership educators in the United States, we reanalyze data originally collected to explore caring pedagogies in leadership programs, now focusing specifically on emotional labor, gendered and racialized expectations of care, and boundary-setting as strategies to sustain care. This analytic shift enables us to surface dimensions of the data that were visible but not central in the initial study, particularly the burdens, tensions, and politics of care work.

This study contributes to the growing body of literature on emotional labor in academia and educational leadership in several distinct ways. Whereas most COVID-19-era faculty studies examine emotional labor among faculty in general or within single disciplines (Constantin et al., 2024; Newcomb, 2021; Heath & Holyoke, 2023), our focus on educational leadership educators foregrounds a group whose emotional labor extends across multiple institutional layers: their own programs, their students’ workplaces, and the communities those students serve. By explicitly integrating Hochschild’s emotional labor framework with Tronto’s political theory of care and feminist analyses of gendered and racialized academic labor (N. N. Johnson, 2021; Laurin, 2022; Scolnic, 2024; Tronto, 1993, 2013), this study moves beyond celebratory accounts of care ethics to examine the politics and distribution of care work in leadership preparation. Through secondary analysis, we demonstrate how re-engaging an existing dataset with a new theoretical lens can generate a distinct storyline—shifting from “care as pedagogical good” to “care as labor and risk”—thus enriching understandings of faculty well-being and the structural conditions of leadership education.

By focusing on educational leadership educators, this study contributes to the Special Issue’s emphasis on well-being in educational contexts in three ways. It foregrounds faculty well-being as inseparable from student well-being and organizational health; it highlights emotional labor as central rather than peripheral to leadership preparation; and it argues that calls for “more care” in education must be matched with structural changes that redistribute and support the labor of care, rather than relying on individual faculty good will (Constantin et al., 2024; Heath & Holyoke, 2023; Tronto, 2013).

### 1.2. Conceptual Framework: Emotional Labor

Hochschild’s (1983/2012) *The Managed Heart* introduced emotional labor as the work of managing one’s own feelings and the feelings of others to produce a desired emotional state in customers, clients, or colleagues according to organizational “feeling rules.” Emotional labor can involve “surface acting” (displaying emotions one does not actually feel) or “deep acting” (attempting to evoke or suppress emotions to align inner feelings with required displays). Both forms can be exhausting and alienating, particularly when they conflict with personal values or occur without institutional recognition and support. Subsequent scholarship has extended the concept beyond service industries to professions where the management of emotions is central to effective practice. In health care, for example, nurses engage in sustained emotional labor with patients and families, work that is linked to stress, physiological strain, and complex identity negotiations (Lim et al., 2018; Mariyanti et al., 2025; Matheson et al., 2019; Pan et al., 2019; Topuz et al., 2024). In organizational and leadership contexts, qualitative work has highlighted how leaders’ emotional labor shapes organizational culture, trust, and perceptions of effectiveness (Azzahra & Rahmawati, 2024; Dopelt et al., 2021).

In higher education, emerging research suggests that faculty also perform extensive emotional labor: reassuring anxious students, mediating conflicts, absorbing frustration about institutional policies, and delivering “bad news” (grades, program requirements) in ways that protect relationships and maintain engagement (Constantin et al., 2024; Newcomb, 2021). During the COVID-19 pandemic, this labor intensified as faculty became key points of contact for students navigating grief, health fears, caregiving responsibilities, and economic precarity (Curran et al., 2023; Heath & Holyoke, 2023). Educational leadership educators were uniquely situated in this regard, supporting students who themselves were leading schools and colleges through crisis, thereby engaging in multi-layered emotional labor that extended beyond the classroom to students’ professional contexts (Mafratoğlu et al., 2025). We draw on emotional labor theory to conceptualize leadership educators’ work not merely as cognitive or technical instruction but as affective and relational labor. This lens foregrounds questions of whose emotions are managed, under what expectations, and with what personal and institutional consequences.

### 1.3. Gendered and Racialized Emotional Labor in Academia

Emotional labor in academia is unevenly distributed across gendered and racialized lines. Feminist and critical scholarship demonstrates that women faculty, and particularly women of color, are more frequently approached for emotional support, granted responsibility for diversity, equity, and inclusion labor, and expected to provide forms of mentoring and pastoral care that function as institutional “office housework” (N. N. Johnson, 2021; Pereira, 2021; Scolnic, 2024). Although this labor is essential to student success and campus climate, it is often framed as altruistic rather than specialized work and remains undervalued within promotion, tenure, and workload structures. These expectations intersect with persistent inequities in unpaid domestic and caregiving responsibilities, which intensified during the COVID-19 pandemic as schooling and eldercare systems were disrupted (Matheson et al., 2019; Pereira, 2021). Faculty of color additionally report carrying racialized emotional labor through supporting students experiencing racial trauma, serving on diversity committees, and educating colleagues about inequities, frequently without proportional recognition or institutional support (N. N. Johnson, 2021; Newcomb, 2021).

Within educational leadership, gender and race shape expectations of relational responsibility and availability. N. N. Johnson (2021) illustrates how Black women leaders navigate intertwined expectations to be resilient, nurturing, and endlessly present, even when such demands compromise their well-being. Relational and narrative approaches to leadership scholarship further reveal how organizations depend upon this often invisible emotional and relational labor to sustain institutional functioning (Gilstrap, 2007; Smit, 2018). Extending this literature, the present study examines how women and faculty of color in leadership education described intensified caring expectations during the pandemic, including modeling vulnerability, holding space for collective grief, and feeling accountable not only to students but also to the communities those students serve. Interpreting these experiences through scholarship on gendered and racialized academic labor situates participants’ narratives within broader structural patterns rather than individual disposition.

### 1.4. Care Ethics and the Politics of Care

Ethics of care scholarship, rooted in the work of Gilligan (1982) and Noddings (2017), understands care as a relational and context-responsive moral orientation shaped by attentiveness, responsibility, and responsiveness to others. Educational applications extend this perspective by emphasizing that students must experience care in order to develop ethical awareness, relational capacity, and social responsibility (Band & Chokshi, 2025). In educational leadership, relational and qualitative traditions further position care as central to organizational life by highlighting dialogue, mutuality, and ethical responsiveness in leadership practice (Azzahra & Rahmawati, 2024; Gilstrap, 2007; Guerra, 2014; Smit, 2018). This scholarship frames care as foundational to both meaningful learning and humane leadership.

Tronto’s (1993, 2013) political theory of care advances this tradition by situating care within structures of labor, power, and recognition. She conceptualizes care as a set of interconnected practices that include caring about, caring for, caregiving, and care receiving, each carrying distinct ethical responsibilities and vulnerabilities. Tronto argues that institutions often rely on marginalized groups, particularly women and racialized communities, to perform disproportionate caring labor while denying corresponding authority or support. Work in professional care settings illustrates how care can function as both ethical commitment and site of political tension when organizational systems fail to redistribute responsibility or provide adequate resources (Laurin, 2022). Within higher education, emotional labor and relational support are essential to student persistence and institutional functioning yet remain insufficiently valued in workload, evaluation, and reward structures (Constantin et al., 2024; Heath & Holyoke, 2023).

Viewing care as an organizational and political issue rather than solely an interpersonal virtue raises questions about how emotional labor is recognized, compensated, and distributed in leadership education. Celebratory narratives of caring faculty can obscure the institutional conditions that make such care both necessary and unsustainable, shifting responsibility from organizations to individual educators. Earlier analysis of this dataset emphasized caring pedagogy and relational practice (Channing & Wilson, 2023). The present study instead centers the labor, risk, and inequity embedded in caring work. By bringing emotional labor theory into conversation with feminist analyses of academic labor and Tronto’s political ethics of care, this study conceptualizes caring in educational leadership as ethically significant yet structurally contested labor that remains central to leadership preparation while often invisible within institutional accounts of faculty work.

Emotional labor theory, feminist scholarship on gendered and racialized academic work, and political theories of care offer a multidimensional framework for understanding caring practices in educational leadership. Emotional labor theory clarifies how feeling is managed within institutional expectations. Feminist scholarship reveals how this labor is unevenly distributed and frequently hidden. Political care ethics situates care within broader structures of power, recognition, and responsibility. Integrating these perspectives allows this study to reconceptualize care in leadership education as structurally organized labor that is ethically meaningful, unevenly allocated, and institutionally consequential. In doing so, the study positions emotional labor as central to leadership preparation and highlights the political conditions necessary for sustaining equitable cultures of care.

## 2. Methods

### 2.1. Original Study and Dataset

The data analyzed in this article come from a qualitative phenomenological study exploring educational leadership educators’ experiences of work and life during the COVID-19 pandemic, with particular attention to caring practices in their teaching. Nine educational leadership educators from across the United States participated. See Table 1. Participants taught in graduate leadership programs preparing PK–12 and higher education leaders and aspiring leaders.

Semi-structured interviews were conducted via Zoom in 2022, with the “pandemic period” defined broadly to include ongoing effects. Interviews invited participants to describe their teaching and leadership roles, their definitions and practices of care, their experiences of work and life during the pandemic, and their perceptions of student needs and institutional responses. Interviews were recorded, transcribed, and verified for accuracy. The original analysis used a phenomenological approach to identify themes related to caring spaces, relationships, learning objectives, and wellness. The original study received institutional review board approval, and participants provided informed consent for their de-identified narratives to be used in publications.

### 2.2. Secondary Analytic Focus

For the present article, we conducted a secondary analysis of the existing transcripts, focusing on the research question:

Secondary analysis of qualitative data is appropriate when new theoretical lenses or questions are applied to rich existing datasets, provided that participant consent and ethical boundaries are respected. In this case, the emotional and political dimensions of care—particularly the burdens and gendered/racialized patterns of care labor—were visible but not central in the earlier analysis, making them suitable for further exploration.

To maintain conceptual congruence between the dataset’s original purpose and the new research question, we limited our secondary analysis to topics that were already substantively present in participants’ narratives—specifically their descriptions of care, relational work, emotional strain, and boundary-setting during the pandemic. We treated emotional labor as an extension rather than a departure from the original study’s focus on caring pedagogies, ensuring that our interpretations remained anchored in participants’ own meaning-making rather than imposing a post hoc theoretical overlay. Ethical boundaries for reanalysis were maintained by using only de-identified transcripts and by adhering to the scope outlined in the original informed consent, which permitted future analytic use of the data for related scholarly purposes. Consistent with best practices for secondary qualitative analysis, we did not introduce new questions, reinterpret sensitive disclosures outside their intended context, or engage in analysis that would have required additional participant verification.

### 2.3. Data Analysis

We employed a theoretically informed thematic analysis. First, we reread all transcripts with attention to passages where participants:

- Described their emotional responses to students’ needs or institutional expectations
- Talked about “being there” for students, “holding space,” or being “the anchor” or “support coach”
- Mentioned exhaustion, role strain, or difficulties balancing work and family during the pandemic
- Reflected on gender, race, or identity in relation to care or support roles

These segments were coded using both deductive codes (e.g., emotional labor, boundary-setting, institutional support, gendered expectations) drawn from the conceptual framework and inductive codes that emerged from the data (e.g., “therapy session” metaphors, “count on me,” invisible guidance).

Next, we clustered codes into broader themes that captured shared patterns across participants while attending to differences by gender, race, and institutional context. Throughout, we compared our interpretations to the earlier published analysis to ensure that we were advancing a distinct storyline rather than restating prior themes.

Both authors participated in the secondary analysis. We began with a shared deductive codebook derived from Hochschild and Tronto, then coded the full dataset iteratively, expanding the codebook inductively as new patterns emerged. We met regularly to compare coding, discuss interpretations, and resolve discrepancies through consensus, returning to full transcript segments to check interpretations against context. Theme development followed this iterative process, with attention to disconfirming evidence and variation across participants.

### 2.4. Reflexivity and Trustworthiness

Both primary and secondary analyses were conducted by educational leadership educators who also lived through the pandemic while teaching and supporting students. Reflexive memos from the original project documented the researchers’ positionalities as first-generation scholars, queer and Latina faculty, educational leaders, and former PK–12 educators, and these memos were revisited in the secondary analysis to surface assumptions about care and leadership. To enhance trustworthiness, we maintained an audit trail of coding decisions, sought disconfirming evidence for emerging themes, and returned frequently to extended transcript excerpts to avoid over-simplification. While member checking for this specific analytic focus was not feasible given the time elapsed, participants had previously reviewed portions of their transcripts and narrative summaries for accuracy.

Because this study draws on interviews with nine educational leadership educators, the findings are intended to offer interpretive insight into participants’ lived experiences rather than to support statistical or universal claims about faculty emotional labor. Throughout the analysis, we distinguish carefully between what participants described in their narratives and the broader structural interpretations informed by emotional labor theory, feminist scholarship, and political theories of care. References to institutional patterns or systemic dynamics are therefore presented as analytic readings grounded in participants’ accounts and existing scholarship, not as claims of generalizability across higher education contexts. This positioning reflects qualitative phenomenology’s emphasis on depth, meaning, and situated understanding.

Reflexive attention was particularly important given the secondary nature of the analysis. The original study centered care as a pedagogical and relational good enacted by leadership educators during the COVID-19 pandemic. In the present analysis, we intentionally shifted the interpretive lens to examine care as emotional labor shaped by gendered expectations, institutional feeling rules, and unequal distributions of responsibility. We approached this shift as an extension of participants’ meaning-making rather than a reinterpretation imposed from outside the data. Interpretations were grounded in passages where participants themselves described exhaustion, role strain, boundary negotiation, and inequities in expectations of care. Reflexive memoing and collaborative analytic dialogue were used to examine how our theoretical commitments shaped what became visible in the data and to ensure that the movement from “care as pedagogical good” to “care as labor and risk” remained ethically accountable to participants’ narratives. These practices support the trustworthiness of the analysis while acknowledging the interpretive role of theory in secondary qualitative inquiry.

## 3. Results

Participants’ narratives revealed deep commitments to caring for students and sustaining learning during a time of overlapping personal and professional crisis. At the same time, they made visible the emotional costs of that care and the ways it was shaped by gendered and racialized expectations and uneven institutional support. For clarity, we refer to participants by pseudonyms: Dr. Elena Ruiz, Dr. Beatrice Carter, Dr. Lauren Price, Dr. David Kim, Dr. Aisha Thompson, Dr. Megan Foster, Dr. Thomas Greene, Dr. Rosa Miles, and Dr. Claire Jensen. Five interrelated themes capture their experiences of emotional labor during the pandemic.

### 3.1. Being “The Anchor” in a Time of Crisis

Across interviews, participants described feeling responsible for providing stability when, as Dr. Carter put it, their students’ “lives were turned upside down.” Many of their graduate students were already serving as “superintendents, administrators … curriculum directors,” simultaneously managing schools, families, and communities under crisis conditions. In this context, Dr. Ruiz explained that she “really tried to make space for grace … if you had a family member or if a student … came down with COVID … I was going to be flexible,” creating “alternative ways of showing proficiency” and “increas[ing] communication” so students knew “if something is happening, let me know so that I can be aware.”

Anchoring students also meant maintaining predictable structures when almost everything else was in flux. Dr. Ruiz noted that “our classes are always in the evenings, since our students work during the day,” and she worked to preserve that routine so students could “count on that time” even while their workdays and home lives were chaotic. Similarly, Dr. Greene reflected that in the midst of the crisis, success sometimes meant recalibrating expectations: “If you make it through, you come to the other side, then you’re … doing fine,” he told students, and if they could “pass your classes … learn[ed] something that you can take … and move forward, then you’ve done successful in a pandemic.”

For Dr. Jensen, who coordinated a principal preparation program that had already moved to fully online, synchronous instruction before COVID-19, the pandemic did not radically change her teaching modality. She emphasized that “we were already … moving along fine” on Zoom, and “it’s not like we had to make adjustments” to the platform. Yet the emotional context of her students’ work changed dramatically. She knew they were teaching all day in schools facing “teacher shortage[s]” and “substitute shortage[s],” often “not [even] hav[ing] a planning period” because they were constantly covering classes. Anchoring them meant adjusting timelines and expectations while insisting that leadership work remained urgent: “I try to be accommodating … but I don’t ever want to let up on my expectations,” she explained. She repeatedly told students that, despite disrupted assessments, “you still have to teach the standards,” and that looking at whatever data were available—“previous data” if necessary—was part of leading schools through crisis rather than pausing that work until conditions normalized.

Several participants described their courses as emotional as well as academic anchors. Dr. Foster recalled that her course had become “a place where we could be exhausted together,” where students could show up saying, “I’m tired,” and she could respond, “me too … let’s talk about it and also let’s think about what this means for your leadership.” She intentionally “created extra space to share where they were in the moment, mentally, physically, emotionally” so class could function as “a touchpoint” rather than another pressure. Dr. Ruiz similarly spoke about intentionally creating “spaces to be open and honest and vulnerable where everybody can learn from each other,” linking that classroom climate to the kinds of environments she wanted her students to later build for “the children… and the families that they are serving.”

This anchoring work extended far beyond delivering content. Dr. Kim described making students “first thing on my to do list … during the day,” striving to be “student responsive” even when that meant sifting through “all of the emails that come across my desk” before other tasks. He refused to respond with terse or cryptic messages, instead offering detailed feedback or short audio explanations so students felt “held” and not ignored. From an emotional labor perspective, being “the anchor” required instructors to project steadiness and hope, “creating those same spaces that I want to create in my class,” as Dr. Ruiz framed it even as they navigated the same uncertainty, fear, and fatigue as their students.

### 3.2. Teaching at the Edge of Counseling: Blurred Roles and Invisible Care Work

Participants repeatedly described moments when teaching felt indistinguishable from counseling or coaching. Dr. Miles reflected that during the height of the pandemic, “students come with everything that’s going on in their lives,” which meant her role was not only to “teach content,” but also to ask, “‘How are you doing? How is your family? What’s going on in your school?’” and to “hold space” for whatever emerged. Class discussions routinely shifted from policy analysis to processing grief, fear, and exhaustion.

Students themselves sometimes characterized courses as therapeutic. Dr. Miles shared, “Some of my students have said it almost feels like group therapy.” Similarly, Dr. Greene described how the work extended well beyond scheduled class time: “Sometimes I felt like I was more of a support coach than a professor,” he explained, because students “would text me, ‘Hey, I’m having this issue at work, do you mind if we just chat about it over text?’” These exchanges could range from navigating a conflict with a supervisor to deciding how to respond when “three staff members tested positive in the same week.”

For some participants, their prior training made the blurring of roles feel both natural and heavy. “My background is social work, so once a social worker always a social worker,” said Dr. Thompson. She saw herself as constantly asking, “How can I help?” and thinking about what it meant to “model vulnerability” so that students could say, “I need help.” She described rewriting her syllabus to acknowledge that a student might be “literally sitting in a room where your grandma is dying in the next room” and insisting that this reality had to change how faculty talked about deadlines, participation, and presence.

Much of this care work was invisible in formal workload measures. It happened in “offline and online conversations,” as Dr. Kim put it—unscheduled phone calls, text threads, late-night emails, and brief check-ins before class officially began. It also occurred in pedagogical moments that were emotionally demanding but not formally recognized, such as sitting through “moments of uncomfortable silence” after raising issues of systemic inequity, and then helping students “see the system in a new light” while simultaneously tending to their emotional responses. Participants were still expected to maintain rigorous instruction and research agendas, but they also became, in their own words, “support coach[es],” therapists, and crisis managers for students’ personal and professional lives.

Dr. Jensen’s narrative extended this blurred role beyond her own enrolled students. As both a leadership educator and a parent, she found herself informally coaching her child’s K–12 teacher through virtual instruction, recalling how the teacher struggled with platforms and formats. Dr. Jensen texted and said, “I do this all the time … if you want me to help you with it I’d be happy to,” offering tips drawn from her university teaching. Her account also highlighted the emotional work of watching K–12 schools prioritize “checking in with [students]” over sustained academic instruction and worrying, as both mother and educator, that “there was a missed opportunity” to teach deeply while students were “captive” online. Even when she framed her orientation as “more the problem-solver” than “touchy-feely,” the labor of constantly asking “what are we going to do about it?” as schools floundered constituted a form of emotional and moral engagement that extended the boundaries of her professorial role.

Several faculty noted that the emotional stakes made it harder to disengage at the end of the day. Dr. Price observed that she and her colleagues “were carrying students’ stories home” and replaying them at night. When one student confided that she was teaching from a parked car because her children were quarantined inside a crowded house, Dr. Price said, “There’s no way to un-hear that. You show up differently from that point on.” The emotional labor of “showing up differently” rarely appeared on annual reviews, yet it structured how participants organized their days, their energy, and their sense of responsibility.

### 3.3. Gendered and Racialized Expectations of Care

Although all participants described caring for students, women and faculty of color often narrated a particularly intensive and relational form of care. Dr. Thompson began by situating herself explicitly: “So, I am … a Black woman … I come from a culture where we’re always thinking about community and collective.” She explained that her approach to teaching is inseparable from that identity and from her social work background, noting, “I’m always thinking, how can I help?” Her students, many of whom were also educators and leaders of color, reacted strongly to the kind of care she practiced.

In one semester, Dr. Thompson redesigned her syllabus to “acknowledge individuals’ needed resources” and to signal that “it was okay to be vulnerable in the class.” She spelled out that cameras could be off, that deadlines were flexible in crisis, and that it was acceptable to say, “I can’t do this this week.” A teaching assistant later told her, “I just need you to know everybody’s not doing this,” emphasizing that students “really appreciate this.” For some, it was the first time they had seen a professor “model vulnerability” and explicitly validate their need for support. Dr. Thompson understood this as part of a broader racialized expectation that Black women would “hold it all together” for students, even when the institution did not formally recognize that work.

Women faculty also talked about extending care through expanded availability and emotional presence. Dr. Ruiz framed her purpose as to “care enough about them to try and tell them the truth, make them see uncomfortable realities,” and to connect those realities “to the stories of the students that they work with in their communities.” She wanted her students, many of whom were already school or district leaders, to leave her class “more knowledgeable,” “more empathetic,” and “more reflective,” and to understand that “their story isn’t everybody’s story” and that “each human has value.” For her, caring for graduate students could not be separated from caring “about the children that they are serving, and the families that they are serving,” and wanting “those kids and those parents to be able to realize their dreams.”

These narratives highlight how care was often framed in familial or communal terms. Dr. Foster described feeling responsible not only for individual students but for “the classrooms, buildings, and communities they go back to.” When she spent extra time helping a student leader think through options for supporting burned-out teachers, she saw it as “caring downstream” for those teachers’ students as well. Dr. Miles similarly reflected that she “doubled down” on checking in with students who were first-generation, working-class, or students of color because she knew they were “carrying extra loads” both on campus and at home.

Dr. Jensen’s account complicated common assumptions about what caring leadership must look like. She did not describe herself as especially “touchy-feely,” instead saying she was “more the problem solver”—someone who listens to “what I’m experiencing” and moves quickly to “what are we going to do about it?” For her, caring for future leaders meant understanding their stressors—teacher shortages, lack of planning periods, disrupted data—and then insisting on practices she believed were essential for equal access, such as strong response-to-intervention systems and careful use of student performance data. While she did not explicitly frame this as gendered, her narrative illustrates another way women in leadership education are expected to carry responsibility: not only being understanding, but also holding the line on rigor and school improvement in a context that tempted many to lower expectations.

Even when participants did not explicitly label their care as gendered or racialized, their stories made those dynamics visible in who spoke about “model[ing] vulnerability,” “fighting for” marginalized students, or “doubling down” on emotional support in the face of institutional neglect. Women and faculty of color often found themselves asked to serve on multiple diversity and equity committees, mentor students from underrepresented backgrounds, and “be the person students go to when they’re hurting,” as Dr. Thompson put it. This convergence of care, equity work, and representation intensified the emotional labor they carried and shaped how they experienced their roles as leadership educators.

### 3.4. Competing Care Obligations Across Work and Home

The emotional labor of supporting students was layered on top of participants’ responsibilities to their own families and communities. Dr. Ruiz described how, when the pandemic began, she was already teaching online, but “all of my children and my husband were now in the house as well.” What had been a quiet workspace turned into “six people in the house, like learning online and then my husband working and taking meetings and me doing that as well, and trying to find space… and really quiet to where I can focus.”

She noted that this shift added roles she “didn’t necessarily have during the day” before: “Where I would take care of myself throughout the day now I’m doing lunch and breakfast … and then making sure that they’re getting online, that they’re focusing online, in addition to me also doing my online work and grading and reading dissertations.” The emotional and logistical labor of caring for children, managing a partner’s remote work, and supporting graduate students all converged in the same physical and temporal space.

Other participants echoed this blurring of work and home. Dr. Carter described the emotional toll of not being able to see her adult children in person, noting “these long grueling spans of time, where … I couldn’t invite them into the house and that was really hard.” She coped in part by bringing two high-energy dogs into her life, explaining that “with my kids being grown out of the house … the dogs kind of fill the void for me,” and that walking them became a crucial part of her self-care. She also spoke candidly about taking “a bath every night” during the most stressful months because “that was the only way to get my body and brain to come down from the day.”

These accounts underscore that the same educators who were extending flexibility and emotional support to students were also juggling intensified caregiving demands at home. The line between “work time” and “home time” became porous. Dr. Ruiz described early-morning hours as “sacred … before the sun comes up,” “the time where I can do as much as I can to feed into me, so I don’t get bogged down from the day.” After that, she said, “everybody else takes my attention, whether it’s work, husband, kids, you know, just stuff.”

Dr. Jensen did not describe extensive caregiving at home beyond parenting a school-aged child, but she highlighted the way virtual work eroded temporal boundaries: when everything moved through the computer, “your day never stops.” Without a commute or clear shift in setting, she experienced virtual teaching, virtual office hours, and constant email as “one continuous job.” Her comments about needing occasional “brain breaks”—stepping away to do laundry, wash dishes, or exercise—captured how care for self and family was squeezed into the edges of an increasingly uninterrupted workday.

Participants also carried emotional responsibilities to extended family and community. Several spoke about checking in daily with elderly parents, siblings who were essential workers, or friends who had lost loved ones. Dr. Thompson noted that in her community, she was “the one people call when something goes wrong,” a role that did not pause just because her institutional workload intensified. Emotional labor in leadership education thus drew from the same finite reserves of time and energy that participants needed to sustain their families and themselves.

### 3.5. Small Acts of Resistance and Boundary-Setting

Despite strong narratives of obligation and responsibility, participants also described efforts to set boundaries and make care sustainable. Several spoke about rethinking what counted as “enough” and resisting perfectionist expectations in their teaching. Dr. Price described planning her grading so she could “look at student work at home, early in the morning” and deliberately “chunking” large sets of assignments—“if there’s a submission of 16 assignments, it’s four a morning for four mornings”—so that she could meet her own standard of returning feedback “within a week” without burning out.

Others set firmer temporal boundaries around their availability. Faculty acknowledged that, early in the pandemic, they responded to student messages at all hours. Over time, they began to limit that. Dr. Price, for instance, decided that it was acceptable if she did not “immediately respond” in the evening and instead addressed messages between 9 a.m. and 7 p.m., explaining to students that “you don’t have to set yourself on fire to keep everybody else warm, and neither do I.” In her view, modeling boundaries was itself a form of leadership education: “If we say we care about well-being, we have to show them what that looks like.”

Participants also resisted institutional narratives that placed responsibility for care solely on individual faculty. Dr. Thompson recounted how her students’ appreciation for a more humane syllabus, one that acknowledged “individuals’ needed resources” and normalized vulnerability, made her question why such practices were not standard. Her teaching assistant’s comment that “everybody’s not doing this” highlighted the uneven distribution of care work across the faculty and prompted her to push colleagues and programs to reconsider assumptions about rigor and support. Dr. Kim similarly challenged colleagues who dismissed flexibility as “lowering standards,” asking, “Whose standards are we talking about, and who pays the price for us pretending nothing’s changed?”

Dr. Jensen’s form of resistance centered on refusing to let the pandemic become, in her words, “an excuse for a lack of rigor and a lack of teaching.” While extending deadlines and broadening options for completing field experiences, she continued to emphasize data use, intervention, and high expectations, recounting her own experience as a principal whose school went from “school in need” to “school of distinction” by systematically analyzing student performance and reorganizing schedules around targeted instruction. She wanted aspiring leaders to understand that, even when state assessments were disrupted and formative data were incomplete, they still had to “do the best we can” with the information available and “not just… stop doing that assignment since things are different.” For her, maintaining rigor and problem-solving in the face of disruption was itself a way of caring for students and the communities they would eventually lead.

Participants emphasized the importance of caring for themselves and one another. Dr. Ruiz described her early-morning writing and reflection time as “sacred… the time where I can do as much as I can to feed into me, so I don’t get bogged down from the day.” Dr. Carter talked about intentionally cultivating hobbies, exercise, and time with pets as ways to sustain the energy needed to care for students. Dr. Miles described starting class by asking everyone, including herself, to name one small act of self-care they had engaged in that week, such as a walk, a nap, a meal shared with family, as a way of “normalizing that leaders have bodies and limits.”

These small acts (e.g., chunking work, redefining availability, redesigning syllabi, naming self-care as non-negotiable) did not eliminate the burdens of emotional labor. But they signal a shift from purely individualized coping toward a more collective and critical stance: questioning “everybody’s not doing this,” imagining alternative norms for rigor and care, and beginning to design teaching practices and departmental cultures that recognize emotional labor as central to leadership education rather than an invisible, limitless resource.

## 4. Discussion

This study illuminates how educational leadership educators experienced and negotiated emotional labor during the COVID-19 pandemic, extending earlier findings on cultures of care toward a more explicit consideration of the labor, risks, and political conditions that sustain such care (Channing & Wilson, 2023). Rather than treating caring pedagogy primarily as a moral good, the present analysis positions emotional labor as central leadership work, demonstrates how this labor is unevenly distributed along gendered and racialized lines, and traces the paradox through which care is simultaneously sustaining and depleting for faculty. These interpretations are grounded in the narratives of nine educators and are offered as situated qualitative insights rather than generalized institutional claims. At the same time, the patterns evident in participants’ experiences resonate with and extend scholarship on emotional labor in higher education (Constantin et al., 2024; Heath & Holyoke, 2023; Newcomb, 2021), particularly by centering educational leadership educators whose work lies at the intersection of teaching, leadership formation, and crisis-responsive professional preparation.

### 4.1. Emotional Labor as Leadership Work

Participants’ accounts affirm Hochschild’s (1983/2012) conceptualization of emotional labor as the management of feeling within institutional expectations. Leadership educators described serving as anchors during crisis, facilitating discussions that resembled counseling spaces, and responding to students’ professional emergencies outside traditional instructional hours. These practices required projecting steadiness, empathy, and reassurance while managing their own uncertainty and exhaustion. Such narratives align with pandemic-era studies documenting how faculty absorbed student distress while sustaining instruction (Constantin et al., 2024; Newcomb, 2021) and with research describing the invisible emotional load carried by higher education leaders navigating multiple constituencies in crisis (Heath & Holyoke, 2023; Mafratoğlu et al., 2025).

The present findings suggest that emotional labor in leadership education is layered across relational and organizational domains. Educators simultaneously teach about ethical and relational leadership, enact those commitments in pedagogy, and support students who are themselves leading institutions through disruption. In this sense, emotional labor extends beyond classroom climate to shape leadership practice in PK–12 and higher education contexts reached through students (Azzahra & Rahmawati, 2024; Bellibaş et al., 2024; Dopelt et al., 2021; Smit, 2018). Parallel dynamics appear in healthcare research during COVID-19, where professionals functioned as emotional buffers between institutions and communities while maintaining a calm and supportive presence (Austin et al., 2021; Dawar et al., 2021; Munn et al., 2021; Prasad et al., 2021). Interpreted through these converging studies, emotional labor emerges not as peripheral to leadership education but as one of the primary ways leadership is enacted and modeled in practice.

### 4.2. Gender, Race, and the Uneven Distribution of Care

Participants’ narratives also reveal how emotional labor is shaped by gendered and racialized expectations. Women and faculty of color described intensified relational engagement, greater availability beyond formal instructional roles, and stronger internalized obligations to support students and their communities. These experiences mirror longstanding findings that women, particularly women of color, disproportionately perform advising, diversity work, and forms of academic care that remain undervalued in promotion and recognition systems (N. N. Johnson, 2021; Pereira, 2021; Scolnic, 2024). Pandemic-era research similarly documents heavier domestic caregiving burdens and disrupted scholarly productivity among women academics (Krukowski et al., 2021; Matheson et al., 2019). Participants’ descriptions of supervising children’s schooling while teaching graduate leadership courses or supporting students through racialized stress while navigating their own marginalization reflect this convergence of professional and personal care obligations.

Tronto’s (1993, 2013) political theory of care provides a critical framework for interpreting these patterns. Institutions depend upon faculty care to sustain student persistence and program continuity, yet often treat such care as an individual disposition rather than a collective responsibility. Applications of Tronto’s framework in professional care settings demonstrate how ethically meaningful labor can become politically precarious when recognition and resources remain uneven (Laurin, 2022). Within leadership education, faculty of color are frequently positioned as cultural and emotional supports for students, roles that may enhance student belonging while constraining faculty research and advancement opportunities (N. N. Johnson, 2021; Newcomb, 2021). Participants’ commitments to equity and justice therefore function both as pedagogical strengths and as sites where emotional labor and institutional expectation converge (Scolnic, 2024). Documenting this intersection extends scholarship on gendered and racialized academic labor into the domain of leadership preparation, where such dynamics have been acknowledged but less systematically examined.

### 4.3. The Paradox of Care: Supportive and Depleting

A central tension across participants’ narratives concerns the dual nature of care. Relational engagement fostered meaning, professional identity, and alignment with ethics-of-care traditions emphasizing attentiveness and responsiveness in education (Gilstrap, 2007; Laurin, 2022; Smit, 2018). At the same time, participants described compassion fatigue, sleep disruption, and feelings of institutional invisibility. Comparable patterns appear in healthcare and nursing literature, where sustained relational demands combined with limited organizational support contribute to burnout and psychological strain (Austin et al., 2021; Lim et al., 2018; Pan et al., 2019; Topuz et al., 2024; Watson, 2023), including during COVID-19 (Chidi et al., 2024; Munn et al., 2021; Prasad et al., 2021).

Higher education research similarly documents compassion fatigue among faculty whose pandemic teaching centered student crisis response (Constantin et al., 2024). Newcomb (2021) further frames academic emotional labor during COVID-19 as gendered, highlighting institutional reliance on feminized care. Participants’ experiences reflect these tensions. Their empathy and flexibility supported student persistence and leadership continuity, yet this labor was rarely recognized within workload or evaluation structures. Acts of boundary setting, such as limiting email response times or protecting personal time, functioned as modest forms of resistance and sustainability consistent with emerging resilience research (Belin et al., 2024; Co, 2022; Uzzell et al., 2025). However, these strategies also reveal the limits of individual coping in the absence of structural change, reinforcing calls to align institutional rhetoric about care with material recognition and support (Heath & Holyoke, 2023; Tronto, 2013).

### 4.4. Contributions to Scholarship and Practice

This study contributes to emotional labor and educational leadership scholarship by centering leadership educators as a distinct group whose work integrates teaching, program leadership, and crisis-responsive mentorship. Prior studies have examined faculty emotional labor broadly (Constantin et al., 2024; Newcomb, 2021) or leadership primarily at administrative levels (Dopelt et al., 2021; Heath & Holyoke, 2023). The present findings instead illuminate how leadership educators simultaneously teach care, model relational leadership, and support students leading institutions during crisis. These interpretations remain grounded in a small qualitative sample while suggesting avenues for broader inquiry into how care is organized and valued across leadership preparation contexts.

The study also advances theoretical integration by bringing emotional labor theory into dialogue with feminist analyses of academic labor and Tronto’s political ethics of care (N. N. Johnson, 2021; Laurin, 2022; Scolnic, 2024; Tronto, 1993, 2013). This synthesis reframes care as ethically meaningful yet structurally contested labor rather than solely a pedagogical virtue. Methodologically, the secondary phenomenological analysis demonstrates how revisiting qualitative data through a different theoretical lens can reveal previously unexamined dimensions of invisible labor, inequity, and boundary setting (Channing & Wilson, 2023). Connections with research in healthcare, nursing, and resilience further situate leadership educators within a broader ecology of care professions navigating tensions between relational commitment and institutional constraint (Austin et al., 2021; Chidi et al., 2024; Munn et al., 2021; Uzzell et al., 2025; Watson, 2023).

### 4.5. Extending Existing Literature on Faculty Emotional Labor

The findings contribute to theory building by positioning emotional labor as a component of leadership labor within graduate preparation. Emotional engagement shapes how leadership identity, ethical responsibility, and relational practice are taught and modeled. Feminist scholarship is extended by illustrating how gendered and racialized expectations influence not only faculty workload but also the leadership norms transmitted to students. Interpreted through Tronto’s political theory of care, participants’ narratives underscore the need for organizational responses that recognize relational labor, redistribute responsibility, and prepare leaders to engage emotional labor ethically and sustainably. Although derived from a small qualitative sample, these insights contribute to ongoing conversations about justice-oriented leadership preparation and collective responsibility for sustaining cultures of care in higher education.

### 4.6. Implications for Theory Building in Educational Leadership

The findings contribute to theory building by reframing emotional labor as a component of leadership labor within graduate preparation. Emotional labor is not only a response to service expectations but also part of how leadership identity, ethical responsibility, and relational practice are taught and modeled. Participants’ accounts suggest that preparing leaders to navigate uncertainty, trauma, and institutional complexity involves sustained emotional engagement that shapes both pedagogy and professional formation.

The study also extends feminist analyses of academic labor by illustrating how gendered and racialized expectations operate within leadership preparation programs, where women faculty and faculty of color frequently assume intensified mentoring and equity-related responsibilities. These dynamics influence not only faculty workload but also the leadership norms transmitted to students, indicating that theories of leadership formation must account for emotional labor as a mechanism through which institutional values and power relations are reproduced.

The findings support movement toward a more explicitly political understanding of care in educational leadership. Tronto’s framework underscores how institutions benefit from faculty emotional labor while providing uneven recognition or support. Interpreting participants’ narratives through this lens suggests the importance of organizational responses such as equitable workload structures, recognition of relational labor in evaluation processes, and preparation of leaders to engage emotional labor ethically and sustainably. Although derived from a limited qualitative sample, these insights contribute to ongoing theoretical conversations about justice-oriented leadership preparation and the collective responsibility for sustaining cultures of care in higher education.

## 5. Implications

### 5.1. For Educational Leadership Preparation

Leadership preparation programs can no longer treat emotional labor as an invisible or purely personal dimension of faculty work. As this study shows, emotional labor is part of how educational leadership educators “do” leadership—through mentoring, modeling ethical practice, and holding space for students’ uncertainty—and thus needs to be named and theorized explicitly in preparation curricula. Gonzales and Rincones (2013) illustrated how higher education administration itself is an emotional endeavor, and their participatory action research makes clear that administrators’ and educators’ emotional experiences are not incidental but constitutive of leadership practice. Likewise, Puyo (2022) argued that ethical leadership can be understood through intertwined lenses of care, justice, critique, and “heartful” education, underscoring that emotions are central to how leaders enact ethics in institutional life.

Programs should therefore integrate explicit discussion of emotional labor, care ethics, and well-being into syllabi, orientations, and faculty development. This can include inviting students to examine how emotional expectations operate in their own institutions, how those expectations intersect with gender, race, and role, and how they might design more just “feeling rules” as leaders. Such work aligns with scholarship emphasizing the formative role of faculty in shaping early student experiences (Abi-Raad, 2018) and the importance of leadership that fosters creativity and innovation within complex social systems (Braun et al., 2016). For leadership educators, naming emotional labor in course objectives, assignments, and reflection prompts positions it not as informal kindness but as a domain of knowledge and skill essential to responsible leadership.

Creating structured spaces within leadership programs for collective reflection is also critical. Trauma-informed learning communities (LaDuca, 2023) and other professional learning communities can provide venues for leadership educators and students to share strategies, process emotional experiences, and co-develop sustainable practices of care. Such spaces resonate with Velez-Cruz and Holstun’s (2022) findings that faculty self-care, burnout, and compassion satisfaction are deeply intertwined and require intentional, relational support rather than individual coping alone. As N. Johnson et al. (2022) noted in their work on faculty development for online teaching, pedagogical training that attends to modality without attending to emotional labor risks leaving faculty underprepared for the relational and affective demands of contemporary leadership education. Integrating care, emotional labor, and wellness into pedagogical development is thus a necessary step toward aligning leadership preparation with the realities of educators’ and students’ work.

### 5.2. For Institutions and Policy

At the institutional level, this study reinforces calls for universities and colleges to move beyond symbolic appreciation of faculty care and to embed emotional labor into the formal structures that govern academic work. Numerous studies have documented how women and faculty of color shoulder disproportionate service and mentoring responsibilities, often in the form of “invisible” care work that is vital to student success but undervalued in evaluation systems (Hanasono et al., 2019; Zambrana et al., 2015). Harper (2012) further demonstrated how higher education research can normalize racist institutional norms when it fails to interrogate the structures that channel emotional and diversity-related labor toward marginalized faculty. Pittman (2010) showed that women faculty of color often navigate racialized and gendered hostility in the classroom, adding another layer of emotional labor to their teaching.

In this context, the emotional labor described by educational leadership educators—anchoring students during crisis, offering individualized support, and processing institutional decisions—should be recognized in workload models, promotion and tenure criteria, and annual reviews. This includes explicitly valuing roles that demand high levels of emotional investment, such as program coordination, intensive advising, mentoring of underrepresented students, and leadership of equity and inclusion initiatives (Braun et al., 2016; Smith, 2020). Velez-Cruz and Holstun (2022) show that unaddressed emotional demands contribute to burnout and threaten faculty well-being, while The Chronicle of Higher Education and Fidelity Investments’ (2020) national survey documents that many faculty were on “the verge of burnout” and reconsidering their career plans during the pandemic. Institutional policies that continue to treat emotional labor as incidental risk accelerating attrition and undermining the very cultures of care they publicly champion.

Equity-focused monitoring is also essential. Hanasono et al. (2019) demonstrate that service loads and care work are often distributed along gendered lines, while Zambrana et al. (2015) highlight the centrality of mentoring for underrepresented minority faculty and the institutional neglect that often accompanies such mentoring. Institutions should systematically examine patterns in service assignments, advising loads, and informal student support—disaggregated by gender, race, rank, and contract status—to identify and remedy inequities. This work should be coupled with broader efforts to foster inclusive climates, recognizing that perceptions of inclusion and belonging among staff and faculty shape how emotional labor is experienced and shared (Nīmante et al., 2021).

These actions align with care ethics perspectives that frame care as a public good and a collective responsibility rather than an individual moral obligation (Tronto, 1993, 2013; Hlengwa & Leibowitz, 2017). Treating care as an institutional responsibility means providing accessible mental health resources for faculty, clarifying expectations around availability, protecting faculty who set reasonable boundaries, and resourcing leadership structures that support rather than offload emotional labor. In short, institutional leaders must view emotional labor not only as “soft” work but as central to student retention, performance, and organizational health (Vogt, 2008; Braun et al., 2016).

### 5.3. For Faculty Practice

For individual faculty members—particularly those in educational leadership—the findings suggest a set of practical strategies for sustaining care without sacrificing personal well-being. As participants in this study modeled, establishing intentional boundaries around availability is crucial. Communicating clear expectations about response times, modes of contact, and the purposes of different kinds of meetings can help faculty offer meaningful support while protecting time for scholarship, family, and rest (Velez-Cruz & Holstun, 2022). Such boundary-setting is not a rejection of care but a way of ensuring that care remains sustainable over time.

Collaboration among colleagues can mitigate feelings of isolation and prevent the concentration of emotional labor on a few individuals. Faculty can work together to design shared mentoring structures, co-facilitate reflective spaces, and rotate responsibilities for high-intensity relational tasks. LaDuca’s (2023) work on trauma-informed learning communities suggests that when educators engage collectively in reflective practice, they are better able to integrate care and trauma awareness into their pedagogy without bearing the emotional load alone. Gonzales and Rincones (2013) similarly show how collaborative, participatory approaches can surface the emotional dimensions of administrative work and open space for shared problem-solving.

Faculty can use their roles in senates, unions, and departmental governance to reframe emotional labor as an institutional issue rather than a private burden. Hlengwa and Leibowitz (2017) call for integrating ethics and care into conversations about quality in educational development; Puyo (2022) urges leaders to weave care and critique together in reimagining educational practice. Building on such work, faculty advocacy can press for policies that recognize care and service, address inequitable labor distributions, and align rhetoric about well-being with material support.

Faculty practice must include care for self as a legitimate professional priority. Velez-Cruz and Holstun (2022) emphasize that self-care, burnout, and compassion satisfaction are interrelated outcomes of the same working conditions. Engaging in routines that protect sleep, health, reflection, and connection—whether through early-morning writing, exercise, spiritual practice, or time with family and pets—should be understood not as optional “extras” but as integral to sustaining the emotional labor that leadership education demands.

## 6. Limitations and Future Research

This study is based on a small, non-random sample of nine educational leadership educators in the United States. Because participation was voluntary and centered on conversations about care and teaching during the COVID-19 pandemic, the sample may overrepresent faculty who are particularly reflective about or invested in caring pedagogies. Educators whose approaches are more transactional, those who compartmentalize emotional work, or those who have already left the profession due to burnout or hostile climates (Pittman, 2010) are likely underrepresented. As with most qualitative research, the aim here is not statistical generalizability but rather analytic insight into how emotional labor was experienced within a specific professional community and historical moment.

The sample composition also imposes natural limits on transferability. Participants were drawn from educational leadership programs and primarily from graduate-level teaching contexts. Faculty in other disciplines—or in institutional settings such as community colleges, regional universities, minority-serving institutions, or international contexts—may encounter different emotional expectations, support structures, and constraints. In addition, although this analysis attends to gender and race, the small sample size prevents definitive claims about intersectional patterns; the gendered and racialized dynamics identified here should be treated as suggestive, pointing to the need for further investigation with larger and more diverse samples.

As a secondary analysis, this study is further constrained by the boundaries of the original dataset. We were limited to the questions and narrative prompts used in the initial study and could not return to participants for elaboration, clarification, or follow-up questions tailored specifically to emotional labor. Thus, the findings are shaped by what participants chose to disclose in the original interviews. While the dataset was rich, the secondary nature of this analysis means that some dimensions of emotional labor—particularly those that participants did not explicitly link to care—may remain underexplored.

Researcher positionality also influenced the analytic process. As educational leadership educators who taught during the pandemic, we share aspects of participants’ professional context and affective landscape. This positionality provided valuable insight into the emotional textures of teaching and leadership work; however, it also required intentional reflexivity to avoid overidentification with participants’ narratives. Analytic memos, team-based coding, and an audit trail were used to mitigate the influence of our own experiences, yet we acknowledge that our interpretations are shaped by our embeddedness in the field.

Future research should broaden the sample and diversify methodological approaches to deepen understanding of emotional labor in leadership education. Mixed-methods studies that pair qualitative interviews with surveys of emotional labor, burnout, job satisfaction, and compassion satisfaction could illuminate relationships between emotional labor and faculty outcomes across institutional types (Velez-Cruz & Holstun, 2022; The Chronicle of Higher Education and Fidelity Investments, 2020). Comparative research examining leadership educators alongside faculty in other disciplines would further clarify which emotional labor dynamics are unique to leadership preparation and which are shared across the academy.

Incorporating student perspectives is another important direction. Students’ views on faculty emotional labor could shed light on how caring practices influence engagement, persistence, and developing conceptions of leadership, particularly in early encounters with graduate education (Abi-Raad, 2018; Vogt, 2008). Research might also include administrative staff, who occupy key relational roles in academic programs and whose perceptions of inclusion and care can reveal broader institutional climates (Nīmante et al., 2021).

Methodologically, future scholarship could follow Gonzales and Rincones (2013) in using participatory action research, visual methods, or narrative inquiry to capture the emotional textures of leadership work. Longitudinal designs are also needed to examine how pandemic-era commitments to flexibility, care, and well-being evolve over time (Newcomb, 2021; Smith, 2020), including whether such practices are sustained, abandoned, or reconfigured as acute crisis conditions recede. Research should examine how leadership development initiatives (e.g., faculty development for online teaching (N. Johnson et al., 2022), trauma-informed communities of practice (LaDuca, 2023), or ethics-of-care-centered leadership training (Puyo, 2022)) shape faculty members’ experiences of emotional labor and their perceptions of institutional support.

## 7. Conclusions

Educational leadership educators in this study navigated the COVID-19 pandemic by engaging in extensive emotional labor: anchoring students during institutional and personal upheaval, hosting “therapy-like” conversations about crisis and loss, and modeling care and ethical decision-making while simultaneously managing their own family and professional responsibilities. Their narratives portray care as a pedagogical strength and a core dimension of leadership practice, but also as a site of vulnerability when it is unevenly distributed along gendered and racialized lines and inadequately recognized by institutions.

Viewed through Hochschild’s framework of emotional labor and Tronto’s political theory of care, the findings underscore that caring in leadership education is not simply an individual virtue; it is labor embedded in organizational structures and power relations. Feminist and critical scholarship on academic labor shows that this work is frequently concentrated on women and faculty of color and is often invisible within conventional measures of productivity and merit (Harper, 2012; Hanasono et al., 2019; Zambrana et al., 2015; Scolnic, 2024). This study extends those insights to the context of educational leadership preparation, highlighting how leadership educators’ emotional labor operates at multiple levels, such as the classroom, program, and profession, and shapes not only their own well-being but also the leadership practices of the educators and administrators they prepare.

The implications are clear: sustainable cultures of care in higher education cannot rely on individual faculty goodwill alone. Leadership preparation programs must explicitly teach about emotional labor and integrate care ethics and well-being into curricula and faculty development. Institutions must recognize, redistribute, and support emotional labor through policies, workload models, and evaluation systems that treat care as central to academic work and organizational health. Faculty, in turn, need both personal strategies and collective avenues for boundary-setting, mutual support, and advocacy.

Attending to emotional labor is, thus, not only a matter of fairness to educational leadership educators; it is central to modeling the kind of ethical, caring, and sustainable leadership that future educational leaders will need as they confront ongoing and emerging crises in schools and colleges. By bringing emotional labor to the foreground, naming it, theorizing it, and designing structures that support it, higher education can move closer to realizing its espoused commitments to equity, inclusion, and care.

## Figures and Tables

**Table 1:** Participant Demographics.

Pseudonym	Gender (Self-Identified)	Race/Ethnicity (Self-Identified)
Dr. Elena Ruiz	Woman	Hispanic/Person of Color
Dr. Aisha Thompson	Woman	Black, Non-Hispanic
Dr. Rosa Miles	Woman	Black, Non-Hispanic
Dr. Beatrice Carter	Woman	White/Non-Hispanic
Dr. Lauren Price	Woman	White, Non-Hispanic
Dr. Megan Foster	Woman	White, Non-Hispanic
Dr. Claire Jensen	Woman	White, Non-Hispanic
Dr. David Kim	Man	White, Hispanic
Dr. Thomas Greene	Man	Black, Non-Hispanic

## Data Availability

The data presented in this study are not publicly available due to privacy and ethical restrictions.
